# Role of TGF-β1 haplotypes in the occurrence of myocardial infarction in young Italian patients

**DOI:** 10.1186/1471-2350-9-13

**Published:** 2008-02-29

**Authors:** Francesca Crobu, Luigi Palumbo, Erica Franco, Serena Bergerone, Sonia Carturan, Simonetta Guarrera, Simone Frea, Gianpaolo Trevi, Alberto Piazza, Giuseppe Matullo

**Affiliations:** 1 Department of Genetics, Biology and Biochemistry, University of Turin, Via Santena, Turin, Italy; 2 Department of Internal Medicine, University of Turin, Cardiology Division, San Giovanni Battista Hospital, Corso Bramante, Turin, Italy; 3 Department of Clinical and Biological Sciences, University of Turin, c/o A.S.O. S. Luigi Gonzaga, Regione Gonzole, Orbassano, Italy; 4 Section of Epidemiology, ISI Foundation (Institute for Scientific Interchange), Villa Gualino, Turin, Italy

## Abstract

**Background:**

Transforming growth factor beta 1 (TGF-β1) gene play an important role in the acute myocardial infarction (AMI), however no investigation has been conducted so far in young AMI patients.

In this study, we evaluated the influence of TGF-β1 polymorphisms/haplotypes on the onset and progression of AMI in young Italian population.

**Methods:**

201 cases and 201 controls were genotyped for three TGF-β1 polymorphisms (G-800A, C-509T and Leu10Pro). The main follow-up end-points (mean follow-up, 107 ± 49 months) were death, myocardial infarction or revascularization procedures.

**Results:**

Significant risk factors were smoking (p < 10^-4^), family history for coronary artery disease (p < 10^-4^), hypercholesterolemia (p = 0.001) and hypertension (p = 0.002). The C-509T and Leu10Pro polymorphisms showed significant differences (p = 0.026 and p = 0.004) between cases and controls.

The most common haplotypes revealed a possible protective effect (GCT, OR 0.75, 95% CI 0.57–0.99, p = 0.042) and an increased risk of AMI (GTC, OR 1.51, 95% CI 1.13–2.02, p = 0.005), respectively.

No statistical differences were observed in genotype distribution in the follow-up study between the two groups: 61 patients with subsequent events (13 deaths) and 108 without events.

**Conclusion:**

Even though our results need to be further confirmed in larger studies, this is the first study reporting on a possible role of TGFβ1 common haplotypes in the onset of AMI in young patients.

## Background

AMI at young age (< 45 years) is a multifaceted disorder characterized by low mortality rates, less extensive coronary artery disease (CAD), good residual left ventricular function, and a favourable prognosis [1]. In addition, young patients show lower occurrence of diabetes and hypertension, but in contrast family history of CAD, hypercholesterolemia, and smoking are more frequent [2].

Genes regulating vascular function, lipid metabolism, coagulation, fibrinolysis and inflammation factors have been only partially investigated in AMI at young age, reporting controversial results [3]. Some polymorphisms in prothrombin gene seem to be associated with increased risk of AMI at young age and Factor VII Leiden polymorphisms seem to be protective, whereas contrasting results have been reported for the polymorphism G894T in the nitric oxide synthase (eNOS) gene and polymorphism C677T in the 5,10-methylenetetrahydrofolate reductase (MTHFR) gene.

A recent promising trend of research was the study of genes involved in inflammatory response related to CAD and atherosclerosis [4]. However, the role of TGF-β1 genetic variants in the occurrence of AMI at young age was not investigated so far.

TGF-β1 is a cytokine expressed by a broad variety of cells, including platelets, connective, haematopoietic and endothelial tissue cells [5]. TGF-β1 is the most abundant isoform of the TGFβ family, and it is a multifunctional cytokine regulating cell growth, differentiation and matrix production [6]. TGF-β1 has been found to enhance the expression of mRNA encoding endothelin in vascular endothelial cells [7] and the synthesis of extracellular matrix components such as collagen, fibronectin, and proteoglycan [8].

Recent evidence suggests that pathological misregulation of the transforming growth factor-β (TGF-β) pathway could be implicated in the development of several major diseases, including myocardial infarction [9], fibrotic disease [10], and inflammation processes [4]. In particular, higher levels of serum or plasma TGF-β1 have been observed in subjects with hypertension, in association with cardiac and renal disease [11,12] or in dilated cardiomyopathy [13], both exacerbated by smoke, although leading to conflicting conclusions.

Even if a clear causative role of TGF-β1 was not demonstrated, changes in the structure and activity of TGF-β1, caused by genetic variants, could influence the biologic processes involving the protein.

We selected three TGF-β1 variants, two of them (G-800A and C-509T) being in the promoter region, near to the consensus DR1 or DR5 nuclear hormone receptor binding sites, crucial for influencing the TGF-β1 synthesis. The third selected polymorphism Leu10Pro (T > C transition) is located into the peptide signal sequence and could interfere with its activity by influencing the transport of the preprotein to the endoplasmic reticulum [14].

In the present study we evaluated the potential role of the above genetic variants in the occurrence and the prognosis of AMI at young age.

## Methods

### Patients and controls

The sample of cases was composed by 201 patients (age 40 ± 4 years), who satisfied the World Health Organization criteria for the diagnosis of myocardial infarction [15], and who were consecutively admitted between 1992 and 2005 to the Coronary Care Unit of the University Cardiology Division of St. Giovanni Battista Hospital of Turin [16].

The 201 patients were matched with healthy control subjects, recruited by General Practitioners, by sex, diabetes status and age (± 1 year). The study complies with the Declaration of Helsinki and was approved by the Inter-hospital Ethical Board, San Giovanni Battista/C.T.O./C.R.F./Maria Adelaide hospitals (President of the Ethical Board Prof. Alessandro Pileri). The subjects gave informed consent and no sex-based or ethnic-based differences were present, although most of them are of Caucasoid origins.

Information on the following conventional risk factors were collected: age, sex, body mass index, smoking habits, history of hypercholesterolemia (total cholesterol levels > 220 mg/dl or use of hypocholesterolemic drugs), diabetes status, hypertension treatment [17], personal and family history of CAD.

A positive family history was considered if the patient had a first-degree relative with CAD at the age of ≤ 55 years for men or at ≤ 65 years for women.

Patients with ascertained congenital hypercoagulable status, with a proved disease limiting life expectance or with declared cocaine abuse were excluded. Control subjects with CAD and any neoplasm, cardiomyopathy or severe illness limiting life expectance or refusing consent were excluded.

Follow-up (mean 107 ± 49 months) was possible until 2005 for 169 cases (84% of the initial population study), and was performed investigating new hospital admissions, performing telephone interview of patients and periodic ambulatory visits.

Endpoint events considered were death, new myocardial infarction and revascularization procedure (angioplasty or coronary artery bypass graft surgery) that occurred after discharge from the index event.

### Laboratory analyses

Blood samples were collected ≥ 6 hours after fasting and 3 months after the index AMI event to avoid influence of acute event on lipid parameters. Total cholesterol, triglycerides, high-density lipoprotein and low-density lipoprotein cholesterol, apolipoprotein B, and apolipoprotein A1 were measured by conventional methods of clinical chemistry.

Genomic DNA was extracted from 200 μl of peripheral blood lymphocytes according to a standard salting-out method [18]. For the 3 polymorphisms, DNA were amplified by Polymerase Chain Reaction and digested with specific restriction endonucleases, as previously reported [19].

### Statistical analysis

We calculated allele and genotype frequencies of the G-800A, C-509T and Leu10Pro polymorphisms by direct gene counting; the chi-square statistics was used to test for Hardy-Weinberg equilibrium.

Haplotype reconstruction and their frequencies were estimated from genotypes data by using a Bayesian statistical method based on the coalescence theory, implemented in the PHASE package version 2.0 (default settings were used).

Linkage disequilibrium parameters (D' and r^2 ^statistics) were calculated by the Haploview version 3.31 software.

A Pearson chi square test or Fisher exact test when appropriate (a P value ≤ 0.05 was considered significant) were performed to compare the distribution of the patients and control for each polymorphism and for each risk factor, both in the acute and follow-up study.

A logistic regression analysis was performed to calculate Odds Ratio (OR) and 95% Confidence Intervals (95% CI), and adjusting genetic risks for smoking habits, family history, hypercholesterolemia and hypertension. With 201 cases and 201 controls, a power of 80% and α = 0.05 we are able to detect an increase in OR = 1.7–2.24 depending on the allele frequencies of the three polymorphisms.

Cox regression was also performed for the follow-up analysis taking into account the time variable. All the analyses were performed by the statistical package SPSS v.13.0.

## Results

Prevalence of classical risk factors among cases and controls are reported in Table 1.

**Table 1:** Prevalence of main conventional risk factors in cases and controls.

Risk factors		Cases N (%)	Controls N (%)	Total N (%)	P value
Smoking	Yes	148 (79.1)	74 (59.7)	222 (71.4)	< 10^-4^
	No	39 (20. 9)	50(40.3)	89 (28.6)	
					
CAD familiarity	Yes	119 (62.3)	20 (20.2)	139 (47.9)	< 10^-4^
	No	72 (37.7)	79 (79.8)	151 (52.1)	
					
Hypertension	Yes	64 (33.9)	15 (16.3)	79 (28.1)	0.002
	No	125 (66.1)	77 (83.7)	202 (71.9)	
					
Hypercolesterolemia	Yes	107 (54.9)	41 (35.7)	148 (47.7)	0.001
	No	88 (45.1)	74 (64.3)	162 (52.3)	

P value = Fisher exact test.

We have found a statistically significant difference between cases and controls for smoking (79.1% in cases vs 59.7% in controls, p < 10^-4^) and familial history of CAD (62.3% vs 20.2%, p < 10^-4^). Significant differences were observed also for hypertension (33.9% vs 16.3%, p = 0.002) and for hypercolesterolemia (54.9% vs 35.7%, p = 0.001).

We genotyped three TGF-β1 polymorphisms in 201 young patients with diagnosed AMI and 201 matched controls (mean age 40 ± 4 years, range 25–45 years). All the polymorphisms were in Hardy-Weinberg equilibrium.

No statistical difference has been observed in the distribution of conventional risk factors by stratifying cases and controls for genotypes (data not shown).

The genotype frequencies (Table 2) appeared significantly different between cases and controls for Leu10Pro (3 × 2 contingency table, p = 0.005), with a significant increased risk as crude Odds Ratios for the CC vs TT genotype (OR 2.35, 95% CI 1.34–4.12, p = 0.003).

**Table 2:** Allele and genotype frequency distribution among AMI cases and controls.

Polymorphism	Genotype	Cases (%)	Controls (%)	P value	Crude ORs (95%CI)	P value	Adjusted ORs (95%CI)	P value
G-800A	GG	175 (87.1)	168 (83.6)	^a^0.571	1		1	
	AG	25 (12.4)	31 (15.4)		0.774 (0.439–1.366)	0.377	0.645 (0.225–1.849)	0.414
	AA	1 (0.5)	2 (1.0)		0.48 (0.043–5.343)	0.551	0.177 (0.002–2.721)	0.427
	G/A	375/27	367/35	^b^0.290				
								
C-509T	CC	67 (33.3)	80 (39.8)	^a^0.062	1			
	CT	87 (43.3)	92 (45.8)		1.129 (0.729–1.749)	0.586	1.365 (0.604–3.083)	0.454
	TT	47 (23.4)	29 (14.4)		1.935 (1.100–3.406)	0.022	1.943 (0.754–5.003)	0.169
	C/T	221/181	252/150	^b^0.026				
								
Leu10Pro	TT	55 (27.4)	69 (34.3)	^a^0.005	1			
	CT	88 (43.8)	101 (50.2)		1.093 (0.693–1.723)	0.702	1.138 (0.481–2.693)	0.768
	CC	58 (28.9)	31 (15.4)		2.347 (1.338–4.117)	0.003	1.589 (0.610–4.136)	0.343
	T/C	198/204	239/163	^b^0.004				

^a ^Pearson chi square P value of the 3 × 2 contingency table; ^b ^Pearson chi square P value of the 2 × 2 contingency table. Crude and adjusted odds ratios (for smoking habits, family history, hypercholesterolemia and hypertension) and 95% CI are reported.

A borderline significance has been observed for C-509T polymorphism (3 × 2 contingency table, p = 0.06), with an interesting increased risk for the TT genotype vs the wild-type CC homozygotes (OR 1.93, 95% CI 1.10–3.41, p = 0.02). However, after Bonferroni correction only Leu10Pro was still significant.

After adjusting for smoking habits, family history, hypercholesterolemia and hypertension, results for C-509T and Leu10Pro polymorphisms were no more significant (Table 2).

No significant association has been observed for the three TGF-β1 genotypes (Table 3) and for haplotypes (not shown) in the follow-up analysis between the group of 61 patients with a second event after AMI and 108 patients without other events both at univariate and multivariate analysis.

**Table 3:** Genotype frequency distribution among the two follow-up groups (events vs non events).

Polymorphism	Genotype	Events (%)	No events (%)	P value	Crude ORs (95%CI)	P value	Adjusted ORs (95%CI)	P value
G-800A	GG	50 (82.0%)	97 (89.8%)		1		1	
	AG	11 (18.0%)	10 (9.3%)	^a^0.196	2.134 (0.849–5.365)	0.107	2.145 (0.850–5.412)	0.106
	AA	0 (0%)	1 (0.9%)		0.000 (0.000)	1	0.000 (0.000)	1
								
C-509T	CC	19 (31.1%)	40 (37.0%)		1		1	
	CT	30 (49.2%)	39 (36.1%)	^a^0.241	1.619 (0.785–3.342)	0.192	1.839 (0.801–4.226)	0.151
	TT	12 (19.7%)	29 (26.9%)		0.871 (0.366–2.072)	0.755	1.024 (0.425–2.470)	0.957
								
Leu10Pro	TT	14 (23.0%)	32 (29.4%)		1		1	
	CT	26 (42.6%)	45 (43.4%)	^a^0.588	1.321 (0.598–2.916)	0.491	1.434 (0.639–3.215)	0.382
	CC	21 (34.4%)	31 (27.2%)		1.548 (0.670–3.577)	0.306	1.711 (0.728–4.022)	0.218

^a ^Pearson chi square P value of the 3 × 2 contingency table. Crude and adjusted odds ratios (for family history) and 95% CI are reported.

No statistical significant association has been reported for G-800A (p = 0.57). In fact, LD pairwise comparison showed stronger significant values between C-509T and Leu10Pro markers, but lower LD value between these two single nucleotide polymorphisms and C-800A (C-800A/G-509T = 0.02, G-800A/Leu10Pro = 0.03, C-509T/Leu10Pro = 0.59, LD values referred to r^2 ^statistic).

Haplotypes were reconstructed and the difference in their distribution between case and control groups was investigated (Table 4). A total of seven haplotypes, with two of them being the most prevalent, were estimated in both groups.

**Table 4:** Estimated haplotype frequency distribution in case and control groups.

Haplotype	Cases (%)	Controls (%)	Chi square	P values	OR (95% CI)
GCT	167 (41.6)	196 (48.7)	4.143	0.042	0.75 (0.57–0.99)
GTC	164 (40.7)	125 (31.2)	7.863	0.005	1.51 (1.13–2.02)
GCC	34 (8.6)	28 (7.0)	0.695	NS	1.25 (0.74–2.09)
ACT	21 (5.3)	25 (6.3)	0.416	NS	0.82 (0.45–1.49)
GTT	10 (2.6)	18 (4.4)	2.281	NS	0.55 (0.25–1.21)
ATC	5 (1.2)	8 (1.9)	0.651	NS	0.63 (0.20–1.97)
ATT	1 (0.2)	1 (0.4)	0.266	NS	0.48 (0.03–8.48)

Total	402	402			

Haplotype = The haplotypes were defined with polymorphisms in the following order: G-800A, C-509T and Leu10Pro.

Only the two most common haplotypes (GCT and GTC) showed statistical differences between the two groups. The frequency of the GCT haplotype carrying the wild type allele at both C-509T and Leu10Pro polymorphism was higher in the control groups (48.7%) than in cases (41.6%) (OR 0.75, 95% CI 0.57–0.99; p = 0.042). On the contrary, the GTC haplotype, carrying the two variant alleles at the above positions, had a higher frequency in the case group (40.7% vs 31.2% in controls; OR 1.51, 95% CI 1.13–2.02, p = 0.005). Both haplotypes carry the wild-type allele of the G-800A polymorphism.

Out of the 61 patients with subsequent events, we registered 13 deaths. Most of the classical risk factors were not considered in the follow-up study due to the adoption of controlled therapies. Cox regression was also performed in the follow-up phase but no significance was observed (data not shown).

## Discussion

To our knowledge, this is the first study investigating the possible involvement of TGF-β1 gene in the occurrence and the prognosis of AMI at young age, although an association has been already described in older patients [9].

TGF-β1 is involved in many biological processes and misregulations in the TGF-β pathways have been investigated in pathologic states such as cardiac hypertrophy [20], atherosclerosis [21] and inflammation processes [4]. Increased TGF-β1 mRNA expression was also observed in human restenotic lesions [22]; on the other hand, it was postulated that increased expression of TGF-β1 at the artery wall level, by inhibition of vascular smooth muscle cells (VSMC), could consequently inhibit the accumulation of lipid in vessel wall [14].

Various TGFβ1 polymorphisms were so far investigated to ascertain their possible involvement in the occurrence of several cardiovascular diseases [23], and they were selected on the basis of their peculiar localization. Even if the relevance of other polymorphisms, as Arg25Pro, has been ascertained in the cardiovascular studies, in our preliminary study we focused only on some of them.

The G-800A variant, occurring at 800 base pairs upstream the 5' principal transcription start site, is located in a crucial consensus CREB half-site [24]; the A allele could inhibit the binding to the transcription factors CRE-BP, which appeared implicated in the regulation of other TGFβ-gene family elements. As the G-800A, so the C-509T variant is a promoter polymorphism, located in a transcription factor consensus binding site; the T allele could affect the TGFβ1 expression levels [14]. Both polymorphisms are situated close to the consensus DR1 or DR5 nuclear hormone receptor binding sites, known as crucial sequences in the regulation of the TGF-β1 synthesis *in vitro *[24] and *in vivo *[24].

The coding Leu10Pro polymorphism, located in the codon 10 of the gene, fall in the signal peptide sequence, necessary to address the synthesized protein to the endoplasmic reticulum. In the past years, consistent conclusions deriving from the study of these TGF-β1 variants were produced, even if the results appear controversial.

In a study of Yokota et al. [14] it was reported a correlation of the wild type allele of C-509T with low TGF-β1 plasmatic concentration. According to these results, the T allele of C-509T was found associated with higher circulating TGF-β1 levels [24], often related to hypertension, and in association with cardiac disease [12] or to dilated cardiomyopathy [13]. The C-509T polymorphism (and its haplotypes) was also recently found associated with myocardial infarction in men [9], even after adjustment for smoking habits, age, hypertension, diabetes and hypercholesterolemia. This result is in agreement with a possible described correlation between genetic variants in the inflammatory genes and the risk modulation of ischaemic heart disease [4]. Moreover, the Leu10Pro variant [14] was found in association with myocardial infarction (MI) in Japanese patients (OR = 3.5 p < 0.0001), although a previous European study [25] did not show any association between this polymorphism and MI.

We have found that both C-509T and Leu10Pro polymorphisms could modulate genetic susceptibility to juvenile AMI, whereas no significant differences was observed for G-800A, in agreement with some studies [9,14], but not with others [25].

Contrasting results could be partly explained by the heterogeneity of the studies, due both to different prevalence of classical risk factors or to different genetic and ethnic backgrounds.

Our results on C-509T and Leu10Pro polymorphisms seem to support the possible relevance of this gene. In fact Leu10Pro does not change the amino-acidic polarity [25], but it is located in a crucial position [14], as above mentioned.

The relevance of C-509T and Leu10Pro is also strengthened by the haplotype results indicating a possible increased risk of AMI conferred by the GTC haplotype and a protective effect of the GCT haplotype. According to these results the putative "protective" haplotype (GCT) carries both the C-509T and Leu10Pro wild type alleles, whereas the putative "predisposing" haplotype GTC carries the mutated alleles. Moreover the two haplotypes were the most common both in cases and in controls, supporting the idea that common variants/haplotypes could be really important in determining common diseases [26]. Although the above described crucial location of the G-800A variant in the genome sequence, we did not observe a significant association between G-800A and AMI.

The lack of significant results for TGF-β1 polymorphisms/haplotypes in the follow-up analysis seems to suggest that different genetic mechanisms could be responsible for the progression of AMI.

## Conclusion

In conclusion, although it is clear that AMI is a multifactorial disease due to the combination of many genetic and well known environmental risk factors, this is the first study reporting on a significant association between TGF-β1 polymorphisms/haplotypes and the occurrence of AMI at young age.

Although these results warrant further investigation on larger sample size and in-depth biochemical analysis relating, for example, haplotypes to the TGF-β1 serum levels, they indicate also the need to better clarify the role of TGF-β pathway in AMI.

## Competing interests

The author(s) declare that they have no competing interests.

## Authors' contributions

FC participated in the design of the study, carried out the molecular genetic studies, participated in the statistical analysis, drafted and revised the manuscript; LP partecipated in the design of the study, in the clinical evaluation and selection of patients, in the follow-up interviews, in the selection of healthy controls, revised the maniscript; EF partecipated in the design of the study, in the clinical evaluation and selection of patients, in the follow-up interviews, revised the maniscript; SB partecipated in the clinical evaluation of patients, in the follow-up interviews, revised the manuscript; SC participated in molecular genetic studies and revised the manuscript; SG molecular genetic studies and revised the manuscript; SF partecipated in the clinical evaluation of patients, in the follow-up interviews; GPT partecipated in the clinical evaluation of patients, revised the manuscript; AP partecipated in the design of the study, revised the manuscript; GM partecipated in the design of the study, in the statistical analysis, revised the manuscript.

## Pre-publication history

The pre-publication history for this paper can be accessed here:


